# Extracellular vesicle-derived miRNA as a novel regulatory system for bi-directional communication in gut-brain-microbiota axis

**DOI:** 10.1186/s12967-021-02861-y

**Published:** 2021-05-11

**Authors:** Liang Zhao, Yingze Ye, Lijuan Gu, Zhihong Jian, Creed M. Stary, Xiaoxing Xiong

**Affiliations:** 1 Department of Gastroenterology, Renmin Hospital of Wuhan University, Wuhan, China; 2 Central Laboratory, Renmin Hospital of Wuhan University, Wuhan, China; 3 Department of Anesthesiology, Perioperative and Pain Medicine, Stanford University School of Medicine, Stanford, CA 94305 USA

**Keywords:** Exosomes, MiRNAs, MiRs, Inter-cellular communication, GBMAx

## Abstract

The gut-brain-microbiota axis (GBMAx) coordinates bidirectional communication between the gut and brain, and is increasingly recognized as playing a central role in physiology and disease. MicroRNAs are important intracellular components secreted by extracellular vesicles (EVs), which act as vital mediators of intercellular and interspecies communication. This review will present current advances in EV-derived microRNAs and their potential functional link with GBMAx. We propose that EV-derived microRNAs comprise a novel regulatory system for GBMAx, and a potential novel therapeutic target for modifying GBMAx in clinical therapy.

## Background

The bidirectional communication and crosstalk between the gut and brain has been well recognized, termed the “gut-brain axis” [1–3]. Emerging evidence implicates gut microbiota in playing a pivotal role in the bidirectional communication that occurs in the gut-brain axis [4], leading to the more recent concept of the “gut-brain-microbiota axis” (GBMAx). Notably, this tripartite axis is coordinated by classical neuro-immune-endocrine and metabolic pathways [4], however the molecular regulation of GBMAx remains undetermined.

MicroRNAs (miRNAs) are small, non-coding RNA molecules capable of modulating gene expression at post-transcriptional level [5]. As an important intracellular component of extracellular vesicles (EVs) miRNAs can be secreted by and transferred to varied target cells [6]. Acting as a vital mediator of intercellular communication, EV-derived miRNAs have been implicated in microbiome-host communication [7, 8]. This review will present the current advances on EV-derived miRNAs and their functional link with GBMAx bi-directional communication. We propose that EV-derived miRNAs represent a novel regulatory system for GBMAx and a potential therapeutic target to modulate GBMAx function.

## The gut-brain-microbiota axis (GBMAx)

The gut-brain-microbiota axis is composed of the following essential components: (1) the neural network, including central nervous system (CNS) the autonomic nervous system (ANS) and enteric nervous system (ENS); (2) the hypothalamic–pituitary–adrenal axis (HPA); (3) neuroendocrine networks including neurotransmitters, hormone and neuropeptides; (4) gut microbiota and their metabolic products; (5) the gut immune system; and, (6) the intestinal barrier and blood–brain barriers [9–14]. Gut microbiota are considered to be a relatively independent and varied mediator of GBMAx, which interact with other components via several neuroanatomic, neuroendocrine, enteroendocrine**,** neuroimmune and metabolic pathways [15].

## Gut microbiota–miRNA interaction

### miRNA-biogenesis and function

A primary miRNA transcript (pre-miRNA) can be processed by several biogenesis procedures to form the miRISC (miRNA-associated RNA-induced silencing complex) [16–19]. miRISC is then guided to target mRNA by complementary base pairing between the target sequence (TS) of the miRNA (nucleotides 2–8 in the 5’- end of the miRNA) and its target site in the 3’-untranslated region (UTR) of target mRNAs [20, 21]. Target gene expression can be down-regulated by either translational inhibition or mRNA degradation according to the extent of base pairing and the surrounding sequences of the TS [22]. Alternatively, some studies also demonstrate that miRNAs can up-regulate transcription of certain target mRNA [23–25]. Notably, a single miRNA can potentially target multiple mRNA, whereas one mRNA can be potentially targeted by multiple miRNAs, reflecting the complex regulatory function of miRNAs [16]. Recent methodological advances including miRNA profiling and loss-of-function studies enable high-fidelity analysis of bioinformation to better define the dynamic expression and functional link with various cellular process and biological pathways in diverse tissues and from diverse species [18, 26, 27]. MiRNAs have been identified as promising candidates for biomarkers and therapeutic targets in a variety of diseases [28].

### Gut miRNA regulate gut microbiota

In 2016, Liu et al. first profiled miRNA expression patterns within feces and gut luminal contents from mice and humans [7]. Intestinal epithelial cells (IEC) and homeobox gene (Hopx)-positive cells were identified as the major cellular source of fecal miRNAs. In vitro studies with cultured *Fusobacterium nucleatium and Escherichia coli* demonstrated that fecal miRNA could regulate bacterial gene transcripts and growth [7]. Targeted deletion of the miRNA biogenesis enzyme Dicer in mice resulted in imbalanced gut microbiota and exacerbated dextran sulfate sodium- (DSS) induced colitis, which was reversed by fecal miRNA transplantation from wild-type littermates, strongly suggesting a critical role of fecal miRNA in shaping gut microbiota and maintaining intestinal homeostasis [7].

More recent studies support an essential role of gut miRNA in inducing dysbiosis related to various disease states. In ovariectomized (OVX) mice, intestinal and fecal miR155/let-7 g expression were increased and associated with altered gut microbiota and cardiovascular function [29]. In another mouse model of total abdominal irradiation (TAI), the expression level of miR-34a-5p was elevated in small intestine, which closely correlated with composition shifting of gut microbiota, possibly contributing to associated cognitive impairment [30]. Distinct fecal or intestinal miRNA expression profiles and their potential link with disease and the abundance of gut microbiota have been identified in inflammatory bowel disease and colorectal cancer, underlying their potential clinical relevance as biomarkers or therapeutic targets [31, 32].

### Gut microbiota regulate gut miRNA expression

The evidence regarding the impact of gut microbiota on host miRNA expression is primarily derived from miRNA expression profile studies comparing traditional mice with germ-free (GF), or colonized mice. Significant differences in miRNA expression profiles in the colon and ileum was detected between GF mice colonized with gut microbiota from GF mice and specific-pathogen free (SPF) colonized littermates [33]. Fecal miRNA expression patterns also exhibited apparent differences between conventional mice and GF mice [34]. Additionally, fecal miRNA profiles can be deferentially and specially regulated by various colitogenic and non-colitogenic microbiota [34]. The potential target mRNAs of those miRNAs may be involved in regulation of xenobiotic metabolism, intestinal barrier maintainance and regulation of immune system function [33, 34].

Other studies reveal that gut microbiota regulate intestinal miRNA profiles in a highly cell type-specific manner [35]. The miRNA expression patterns of intestinal epithelial stem cell (IESC) are most significantly altered in response to gut microbiota among all intestinal epithelial cell types, with miR-375-3p identified as selectively sensitive to microbiota from IESC [35]. In addition to intestinal miRNA, the expression of fecal miRNA can also be influenced by gut microbiota. Higher abundance of fecal miRNA profiles is detected in GF mice than SPF colonized littermates, and alterations in fecal miRNA expression patterns can be induced by depleting gut microbiota with antibiotic in SPF mice [7, 36].

In vitro studies demonstrate that commensal bacteria induce certain miRNA expression patterns in intestinal epithelial cells or dendritic cells,targeting mRNAs that regulate the innate immune response and barrier function [37, 38]. *Adherent-invasive E. coli* (AIEC), a pathogen with high prevalence in Crohn’s disease, has been shown to up-regulate miRNAs targeting genes responsible for the autophagy response (ATG5 and ATG16L) in mouse enterocytes, which may facilitate AIEC replication and exacerbation of intestinal inflammation [39]. Probiotics including *E. coli Nissle 1917, lactobacilli, Lactobacillus rhamnosus GG, Enterococcus faecium NCIMB 10,415, Enteropathogenic E. coli* have also been shown to modulate miRNAs in intestinal epithelial cells or immune cells thereby altering intestinal immune regulation and barrier function [40–43].

### Gut microbiota regulates brain miRNA expression

A large number of abnormal brain miRNAs implicated in anxiety-like behaviors have been detected in the region of amygdala and prefrontal cortex of GF mice or mice with microbiota depletion by an antibiotic cocktail [44]. Some dysregulated brain miRNAs in GF mice have been shown to be normalized by microbial colonization [44]. Gut microbiota have also been demonstrated to modulate hippocampal miRNA expression associated via kynurenine pathway enzymes which regulate hippocampal development and axon guidance pathway [45, 46]. A more recent report describes that a microbial product, *Bacteroides fragilis* lipopolysaccharide (BF-LPS) can act as a neurotoxin via induction of a series of miRNAs targeting genes that regulate synaptic architecture and deficits, amyloidogenesis, and cerebral inflammatory signaling [47]. Some other microbial metabolites including tryptophan, butyrate, acetylcholine, norepinephrine, serotonin, dopamine may also influence miRNA biology indirectly via regulation of astrocyte function and blood–brain-barrier integrity, or even by altering human behavior via disruption of normal neurotransmitter levels [48]. The gut microbiota—host miRNA interaction is summarized in Tables 1 and 2.

**Table 1:** Gut/fecal miRNA capable of modulating gut microbiota and their function

Gut/fecal miRNA	Function	Disease /experimental model	References
miR155/let-7 g	Cardiovascular function	Ovariectomized mice	[29]
miR-34a-5p	Cognitive impairment	Total abdominal irradiation (mice)	[30]
miR-182, miR-503, mir-17 ~ 92 cluster	Glycan production in recruiting bacteria to tumor	Colorectal cancer (patients)	[31]
miR-199a, miR-223-3p, miR-1226, miR-548ab, miR-515-5p	Disease activity and prognosis of inflammatory bowel disease	Inflammatory bowel disease (patients)	[32]

**Table 2:** Gut or brain miRNA modulated by gut microbiota/microbial products and their function

miRNA	Cell /tissue/organ	Function	References
miR10-a	Dendritic Cell	Innate immune responses	[37]
miR-21-5p	Intestinal epithelial cells	Intestinal epithelial barrier	[38]
miR-30c,miR-130A	Enterocyte	Autophagy response	[39]
miR-203, miR-483-3, miR-595	Intestinal epithelial cells	Intestinal epithelial barrier	[40]
miR -423-5p	Intestinal epithelial cells	Immune responses	[41]
miR-155, miR-223	Colon	Intestinal epithelial barrier	[42]
miR-146a	Intestinal epithelial and monocytic Cells	Intestinal inflammation	[43]
miR-294-5p	Hippocampus	Kynurenine metabolism	[45]
miR-9, miR-34a, miR-125b, miR-146a, miR-155	Neuronal-glial cells	Inflammatory neurodegeneration	[47]

## Extracellular vesicles (EVs)

### EV biogenesis and function

EVs compromise a variety of endogenous membranous-bound nanovesicles released from cells into the extracellular space [49]. EVs can be detected abundantly in bodily fluid and peripheral blood and can be divided into three subtypes according to biogenesis, size, composition, and cargo: apoptotic bodies, micro-vesicles (MVs), and exosomes [49–51]. EVs play a critical role in cell-to-cell communication under both physiological and pathophysiological conditions via transfer of nucleic acids and protein to recipient cells. This delivery system enables intra and inter-species crosstalk including microbiota-host interactions under both physiological and pathophysiological conditions, even without close cellular contact [6].

Exosomes are currently the most well-recognized and described subtype of EVs, characterized by having a diameter of 30–100 nm. They are initially derived from internalization of the cell membrane, which results in accumulation of intraluminal vesicles (ILVs) and formation of multi-vesicular bodies (MVBs) [52]. After fusing with the plasma membrane, the content of MVBs are released into the extracellular space to form mature exosomes. Exosome can be taken up by recipient cells with horizontal transfer of their cargos including DNA, RNA (mRNA, miRNA, non-coding RNA) and proteins [53–55]. Exosomes participate in multiple cellular process related to gene transcription and translation, transcript and protein modifications, protein localization, and key enzymatic reactions [56–59]. MiRNAs have been detected in exosomes derived from cancer cells, virus-infected cells, and mesenchymal stem cells, playing a fundamental role in intercellular communication via transfer of translational control in various physiological and pathophysiological processes [60–70]. There have been four proposed pathways for sorting miRNAs into exosomes: (1) a neural sphingomyelinase 2 (nSMase2)-dependent pathway; (2) a sumoylated heterogeneous nuclear ribonucleoprotein (hnRNP)-dependent pathway; (3) guide dance by the 3’ end of the miRNA sequence; and, (4) mediation by the miRNA-induced silencing complex (miRISC) [71–76]. However, controversy remains on the exact composition of EVs secondary and the presence and abundance of EV miRNA and miRNA carriers [77–81].

### Influencing factor for EV biogenesis

The biogenesis of EV is regulated by a variety of intracellular proteins, enzymes and signaling pathways including: (1) RNA-binding proteins such as hnRNPA2B1 and Argonaute-2; (2) membranous proteins such as Caveolin-1 and Neural Sphingomyelinases; (3) Rab GTPases, ARRDC1, and ESCRT complexes; (4) lipid rafts or membrane lipid microdomains; (5) cytosolic proteins (syntenin) and endosomal enzymes (Heparanase); and, 6) Intracellular calcium-signaling pathways [82–89]. Biogenesic processes can also be modulated by different extracellular stimuli including: (1) viral infection; (2) oncogenic transformation or stresses; (3) hypoxia; (4) alcohol exposure; (5) irradiation; (6) impaired autophagy; and, (7) circulating hormones, which all have important implications in elucidating the pathophysiological mechanisms for development of novel therapeutic targets [90–95].

### EV entrapment of fecal miRNA

In their study on fecal miRNA expression profiles, Liu et al. detected EVs in fecal samples and demonstrated that the most abundant fecal miRNAs were also contained within EVs, suggesting that EVs are the major extracellular source of fecal miRNAs [7]. EVs protect fecal miRNAs from degradation via a phospholipid bilayer comprising membrane proteins of EV which entrapping miRNA [96, 97].

### Brain-derived EVs

Recent studies describe a wide distribution of EV in the CNS, detected in oligodendrocytes, neurons, astrocytes, microglia, choroid plexus, and brain epithelial cells the interface of blood–brain barrier (BBB) and cerebrospinal fluid (CSF) [98–100]. Brain-derived EVs play a key role in cell-to-cell communication involved in neurogenesis, neural development, neuro-inflammation, synaptic communication and nerve regeneration [101–104]. Accumulating evidence suggest that brain-derived EVs, especially exosomes, play an important role in the pathogenesis of neurodegenerative diseases, infectious CNS diseases, neuroinflammation, psychiatric disease and brain tumors [105–111].Their output and cargo can be cell-specific and disease -specific and varied with different events during disease progress, features that provide strong potential for use as a biomarker for CNS disease [108, 112, 113]. Furthermore, several other key features of EVs including stability, low immunogenicity, facility of crossing the BBB, accurate cell targeting and specific delivery make them an attractive candidate for therapeutic delivery vehicles in treating CNS disease [114–116].

MiRNAs have been demonstrated to play an important active biological role within brain-derived EVs from astrocytes, neurons, macrophage/microglial cells, prefrontal cortices cells, glioma cells, glioblastoma cells, and glioblastoma stem-like cells, playing a critical role in neurogenesis, response to stress, virus induced neurotoxicity, schizophrenia and bipolar disorder, brain tumor progress, brain metastasis outgrowth [101, 117–120]. More recent research indicates that brain-derived EVs can be detectable in plasma, and astrocyte-derived exosomes are capable of transferring miRNA to metastatic tumor cells, suggesting that brain-derived EVs may transfer molecular information to tissues remote from the CNS [120–122]. Several recent studies have demonstrated that altered miRNA profiles in brain EVs from Alzheimer's patients, however the mechanisms and clinical significance underscoring these observations remain a focus of investigation [123–125]. Critically, the biological relevance for EV transfer from brain to gut has not been fully elucidated.

### Microbiota-derived EV

Bacterial membrane vesicles, including outer-membrane vesicles (OMVs) derived from Gram-negative bacterium and membrane vesicles (MVs) derived from Gram-positive bacteria, parasites, fungi, mycobacteria, refer to a collection of nano-sized membrane vesicles released from bacteria into the extracellular environment [126, 127]. Bacterial membrane vesicles are currently regarded as microbiota derived-EVs since they share characteristic similarities in size, structure and biological function with EVs derived from mammalian cells [128]. Microbiota-derived EVs can transfer a broad range of cargo including bioactive proteins, lipids, nucleic acids, and virulence factors to neighboring bacteria or host cells (epithelial cells, endothelial cells, immune cells). This bioinformatic transferring plays a critical role in cellular processes for both intra-kingdom (bacteria-bacteria) interactions and inter-kingdom (bacteria-host) communications [129, 130]. The effect of microbiota derived EVs can be effectively differentiated from microbial metabolites or host by evaluating the effect of bacterial free microbiota-derived EVs isolated from bacterial cultures on fecal samples [131]. Recent advances in this field reveal that microbiota-derived EVs exhibit multiple regulatory functions central for bacterial survival and nutrient acquisition, bacterial virulence delivery, host colonization and invasion, microbial interactions, antimicrobial resistance, stress and inflammatory response, endothelial cell adhesion, and systemic inflammatory and metabolic response, which all play key roles in the pathogenesis of diverse infectious and inflammatory diseases [132–139]. Several key features of OMV including size, antigen stability, high immunogenicity, accurate host cell targeting, specific cargo delivery and host immune response make them a promising novel candidate for a vaccine target against bacterial infections, and as targeted drug delivery against cancer and other diseases [140–142]. Recent findings have focused on the modulatory effect of microbiota-derived EVs on intestinal barrier function and the immune response, two important components of GBMAx [143–148]. Furthermore, relevant studies also reveal that microbiota-derived EVs can be released into the systemic circulation and cross the BBB [8, 149, 150]. *Staphylococcus aureus and Helicobacter pylori*-derived EVs have been detected in the brain after oral administration or intramuscular injection via in vivo imaging procedures [151, 152]. Additionally, LPS, a key virulence factor in *porphyromonas gingivalis* outer membrane vesicles has been found in glia and the major cerebral vessels of patients with Alzheimers disease (AD) by immunoblot [153]. It has been hypothesized that microbiota-derived EV may be absorbed into mesenteric veins, carried by the hepatic portal vein and liver, to finally enter the brain via the circulatory system [154]. These data strongly suggest that microbiota-derived EVs may exert a direct effect on the CNS and be an important central modulator for GBMAx.

Small RNA (SRNA) within microbiota derived EV can be internalized by host cells and play an important role in host–pathogen interaction. miRNA-sized sRNA and methionine transfer RNA (tRNA) secreted by bacterial OMV *(periodontal pathogens and Pseudomonas aeruginosa*) have been shown to enter host cells and modulate host immunity [155, 156]. EV-contained miRNA secreted by gastrointestinal nematode has been detected in circulation, which can be internalized by small intestinal epithelial cells and modulate host innate response [157]. Microbiota derived RNA may act as ligands for Toll-like Receptor (TLR) and regulators for host innate immunity [158, 159].

More recent research revealed that OMV may cross the blood–brain barrier and contribute to neuroinflammation and cognitive impairment linked with neurodegeneration disease such as Alzheimer's disease, Parkinson’s disease and dementia. The possible mechanism may involve transfer of small RNA non-coding RNA elements contained within OMV into host cells, thereby regulating host gene expression [160–165].

### EV derived miRNA in metabolic disease

Obesity, Metabolic Syndrome and diabetic mellitus are known risk factors for the development of CNS disorders including cerebrovascular disease, neurodegenerative diseases and dementia. Several lines of evidence have revealed that EV derived miRNA originated from gut microbiota, adipose tissue, steatotic hepatocytes, mesenchymal stem/stromal cells (MSC), and pancreatic islets play crucial role in the pathogenesis of those metabolic disease and associated target organ injury [166–171]. Their role and relevance to GBMAXs and cerebral disease remains an area of active investogatyion. The impact of EV derived miRNA on neurological and metabolic disease are summarized in Table 3.

**Table 3:** The impact of EV derived miRNA on neurological and metabolic disease

EV sRNA	EV origination	Function	References
hsa-miR-23a-3p, hsa-miR-126-3p, hsa-let-7i-5p, hsa-miR-151a-3p (Downregulated)	Plasma	Unknown in Alzheimer's disease	[123]
miR-212 and miR-132 (Downregulated)	Neurally derived plasma EV	Unknown in Alzheimer's disease	[124]
miR-23a-3p, miR-223-3p, miR-190a-5p, miR-100-3p, (Downregulated)	Neurally Derived Plasma EV	Unknown in Alzheimer's disease	[125]
miRNA cargo (periodontal bacteria)	Aggregatibacter actinomycetemcomitans	Neuroinflammation in Alzheimer's disease	[165]
miR-27b,miR-126 miR-130, miR-296	Pancreatic islets	Beta cell-endothelium cross-talk in diabetes	[165]
miR-221-3p (up regulated)	Perivascular adipose tissue	Vascular remodeling in obesity	[168]
miR-1 (up regulated)	Steatotic hepatocytes	Atherogenesis in Non-alcoholic fatty liver disease	[170]
miR-136-3p, miR-4798-5p miR-12,136, miR-222-3p (Downregulated) miR-630, miR-144-3p, miR-143-5p, miR-4787-3p miR-769-5p, miR-8074, miR-181a-5p) (up regulated)	Mesenchymal stem cells	Renal tubular cells senescence in metabolic syndrome	[171]

**Table 4:** The work-flow of the literature review

Topic	References
1.Gut-brain-microbiota axis (GBMAx)	[1-4, 9-15]
2.microRNA( miRNA)	
2.1.miRNA biogenesis	[16-19]
2.2.miRNA function	[20-28]
3.Gut microbiota-host miRNA interaction	
3.1.gut microbiota-gut miRNA interaction	[7, 29-43]
3.2.gut microbiota-brain miRNA interaction	[44-48]
4. Extracellular vesicles (EVs)	
4.1.EV biogenesis	[49-55, 82-95]
4.2.EV function	[60-81, 172-177, 166-171, 187-191]
4.3.Environmental and genetic influence	[178-186]
4.4.EV entrapment of fecal miRNA	[7, 96, 97]
4.5.Brain- derived EVs	[98-125]
4.6. Microbiota derived EVs	[126-165]

## Controversies and challenges

EV derived miRNA has gain great attention in the research of GBMAx. However, controversies and challenges remain in this fields.

### EV classsification and miRNA extraction

The heterogeneity of EV may be far greater than we have recognized previously. A more complex classification system based on EV proteome, nucleic acid distribution and biological function (rather than only 3 subsets mentioned above) has been predicted1 [172–174]. Practical difficulty may exist in extraction of EV-derived miRNA including (1) tedious and costly procedures of ultracentrifugation and density gradient extraction, and purification; (2) lack of standardization with technological platforms and quantitative assays; (3) non-selective enrichment of specific EV subpopulations or differential cellular origins; and, (4) uncoupling from conventional reverse transcriptase quantitative PCR [175–177]. Novel extraction approach and technological improvements are warranted.

### Environmental and human genetic factors

It must be acknowledged that the regulatory system of EV-derived miRNA on GBMAXs is not restricted to EV or miRNAs originating from gut microbiota, gut or brain. Environmental factors (e.g. diet, medications, smoking, environmental contaminants, stress) and human genetics also play a crucial modulatory role in GBMAXs via: (1) secreting miRNA containing EVs; (2) shaping gut microbiota; (3) stimulating microbial metabolic products; and, (4) regulation of miRNA expression and function of the host gut and brain [178–184]. The expression of host miRNA ( fecal miRNA or intestinal epithelial cell-derived miRNA) and its associated function is impacted by these host genetic and environmental factors, which will ultimately modulate the composition of metabolite and function of gut microbiota [34, 185, 186]. Those environmental and genetic factors may be considered as an extension of the EV-derived miRNA system for GBMAXs, and should be taken into accounting novel drug development and therapeutic strategies targeting GBMAx.

### Non-miRNA RNA biotypes and non-vesicle carriers

miRNA is the most studied extracellular RNA but only constitutes a minor composition of RNA biotype in the EV cargo. Other RNA biotypes including small nucleolar RNA (snoRNA), small nuclear RNA (snRNA), long non-coding RNA (lncRNA), Y RNA may be more abundant in EV cargo [187–189]. EV is not the only RNA carrier for miRNA. Non-vesicular miRNAs presenting as ribonucleoprotein complex have been detected in various fluids and circulation, which are becoming candidates for biomarkers and therapeutic targets [190, 191]. The regulatory systems consisting of non-miRNA RNA biotypes and non-vesicle carriers in GBMAx and their relationship with EV derived miRNA should be explored in further study (Table 4).

## Conclusions

**Fig. 1:**
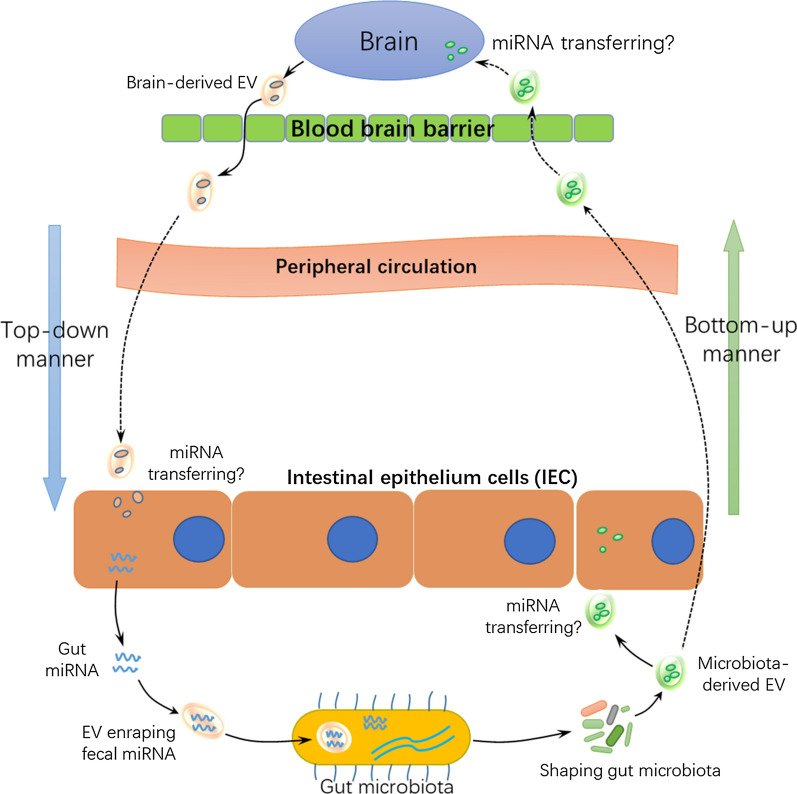
Schematic presentation of EV derived miRNA acting as a novel regulatory system for bi-directional communication in gut-brain-microbiota axis. A proposed regulatory system consisting of extracellular vesicles (EVs) derived from the brain, gut and gut microbiota which modulate bi-directional communication in gut-brain-microbiota axis (GBMAx) via intercellular transfer of microRNAs (miRNAs). Brain-derived EVs may modulate the gut and gut microiota via a “top-down” manner by migrating from brain to gut and regulating the expression of gut miRNAs and fecal miRNAs. Fecal miRNAs entrapped within EVs can enter bacteria and shape gut microbiota via targeting bacterial nucleic acid sequences. Alternatively, or in parallel, microbiota derived-EVs (bacterial membrane vesicles) may modulate the brain via a “bottom-up” manner by crossing the blood brain barrier and exerting a direct effect on the central nervous system. Microbiota derived-EVs can also potentially modulate gut barrier function and the immune response directly

MiRNAs play a potentially critical role in gut microbiota-gut interaction and gut microbiota-brain bi-directional communication. EVs can be derived from brain, gut and gut microbiota, coordinating cell-to-cell communication via transfer of miRNAs. We hypothesize that an EV-miRNA system throughout GBMAx could play a central role in exchange of molecular information among gut microbiota, gut and brain. This EV-miRNA based regulatory system is schematically outlined in Fig. 1. However, current research in this field remains in the early stages. Further investigations should be performed to elucidate: (1) the direct effect of brain-derived EVs on gut and gut micribota; (2) the precise regulatory mechanisms of EV miRNA transfer, and their biological function on GBMAx; (3) the functional link between EV-miRNA and other classical neuro-immune-endocrine pathways. Progress in this field will provide new insight into the comprehensive understanding of GBMAx and help advance the clinical development of novel biomarkers and therapeutic target for the variety of diseases associated with GBMAx imbalance.

## Data Availability

Not applicable.

## Abbreviations

GBMAx: Gut-brain-microbiota axis
EVs: Extracellular vesicles
CNS: Central nervous system
ANS: Autonomic nervous system
ENS: And enteric nervous system
HPA: The hypothalamic–pituitary–adrenal axis
pre-miRNA: Primary miRNA transcript
miRISC: MiRNA-associated RNA-induced silencing complex
IEC: Intestinal epithelial cells
Hopx: Homeobox gene
OVX: Ovariectomized
TAI: Total abdominal irradiation
GF: Germ-free
SPF: Specific-pathogen free
IESC: Intestinal epithelial stem cell
LGG: Lactobacillus rhamnosus GG
BF-LPS: Bacteroides fragilis lipopolysaccharide
MVs: Micro-vesicles
ILVs: Ntraluminal vesicles
MVBs: Formation of multi-vesicular bodies
AD: Alzheimers disease
